# Folic Acid and Endothelial Dysfunction in COVID-19

**DOI:** 10.3390/life16071116

**Published:** 2026-07-04

**Authors:** Maria Macarena Massip Copiz

**Affiliations:** Laboratory of Respiratory Diseases, Buenos Aires C1124AAA, Argentina; mamacarenamc@gmail.com

**Keywords:** COVID-19, SARS-CoV-2, folic-acid, vitamin B9, folate, homocysteine, endothelial-dysfunction, vitamin-B12, long-COVID, PASC

## Abstract

Since 2020, recurrent waves of SARS-CoV-2 infection have persisted globally. Despite the advancements in vaccines and pharmacological treatments, a subset of patients still exhibits an aggressive form of COVID-19 requiring prolonged stays in the intensive care unit (ICU) or experiences major acute infections (or reinfections) and long-term symptoms. Endothelial dysfunction is one of the key events contributing to both the severity of acute COVID-19 and the development of long COVID (LC)/post-acute sequelae of SARS-CoV-2 infection (PASC) syndrome. Since the beginning of the pandemic, the efficacy of nutraceuticals, particularly essential micronutrients, has been investigated as a complementary treatment to prevent disease onset and improve clinical outcomes. One such bioactive molecule is folate (vitamin B9), a member of the B-vitamin family involved in the pathogenesis of multiple diseases, including viral infections and vascular disorders. This review examines the role of folic acid in COVID-19 and its interaction with homocysteine metabolism, which is frequently dysregulated in inflammatory endothelial diseases. It further discusses the potential benefits of folic acid supplementation for the prevention and treatment of COVID-19 in both the acute and long-term phases of the disease, alongside the therapeutic role of vitamin B12 supplementation in LC syndrome.

## 1. Introduction

COVID-19 (coronavirus disease 2019) has raised worldwide concern since it was first reported in December 2019 in Wuhan city, Hubei province, China. It started as an outbreak of viral pneumonia, which was later attributed to a novel coronavirus called SARS-CoV-2 (severe acute respiratory syndrome coronavirus 2) [1,2,3]. On 11 March 2020, the World Health Organization (WHO) declared COVID-19 a pandemic, and the consequences have been devastating around the world. More than 779 million cases and 7.1 million deaths have been officially reported [4], and the real death toll is expected to be three times higher [5].

The mean incubation period for the onset of COVID-19 symptoms has been estimated to be 5.1 days, with most individuals developing symptoms within 14 days after exposure to the virus [6]. Patients can be classified into four different categories according to the severity of the disease: asymptomatic, mild, moderate and severe [7]. The clinical presentation is primarily characterized by fever, cough, fatigue, shortage of breath and the sudden loss of smell and taste [8,9,10,11]. Digestive symptoms such as nausea, vomiting, abdominal pain and diarrhea are also manifested in some patients [12]. Severe cases may present with acute respiratory distress syndrome (ARDS), shock, encephalopathy, myocardial injury, heart failure, coagulation dysfunction or acute kidney injury, which can subsequently progress to multiple organ dysfunction syndrome (MODS) [13,14]. In patients with severe respiratory failure (SRF) requiring intubation and mechanical ventilation (MV), the risk of death is high and can reach 50–60% [15,16,17].

Fortunately, over six years since the beginning of the pandemic, the immunity gained through vaccination and repetitive infections has reduced COVID-19 severity and mortality worldwide. Nonetheless, it continues to result in hospitalizations and fatal outcomes among vulnerable groups, leaving many others suffering the long-term consequences of the infection. Furthermore, evidence suggests that both clinical symptoms and systemic pathology can persist for several years, with reinfection potentially contributing to the accumulation of these effects over time [18,19].

Currently, the incidence of SARS-CoV-2 infection exhibits a biannual seasonality [20,21], characterized by two distinct peaks, although the virus circulates throughout the year. These events can be clearly observed in the US, where continuous monitoring of viral activity in wastewater [22] facilitates the timely identification of pathogens like SARS-CoV-2 and functions as a proactive surveillance system for infectious diseases [23].

As mentioned above, following the peak of the COVID-19 pandemic, there has been a decline in the infectivity and mortality rates of SARS-CoV-2. Nevertheless, society remains vulnerable to the proliferation of novel SARS-CoV-2 variants, such as XFG.1.1 [24], considering that a substantial proportion of individuals experience long COVID-19 symptoms, even after recovery from the acute phase of the disease. Furthermore, significant advancements have been made in diagnostic technologies, treatments, and vaccines against this virus. However, the emergence of novel SARS-CoV-2 variants threatens to reduce the effectiveness of these tools, highlighting the need for continued research and innovation [23]. Consequently, COVID-19 persists as a significant public health concern and continuous updates on SARS-CoV-2-related research are crucial for managing its current endemic status.

As has been extensively described, in severe cases, a dysregulated host immune response and the increased production of inflammatory cytokines may lead to a poor prognosis of SARS-CoV-2 infection [25]. Therefore, patients with this hyperinflammatory condition must be detected quickly, as they suddenly deteriorate [26]. Here, the endothelium plays a significant role in regulating inflammatory processes in the context of COVID-19. Indeed, deaths from COVID-19 result from both ARDS and severe pulmonary vascular disease with important endothelial damage, producing an impairment of lung oxygenation and ventilation [27].

Likewise, the long-term consequences of COVID-19 infection, such as cognitive decline and reduced exercise performance, may also be affected by persistent or residual endotheliopathy [28]. Notably, endothelial complications are not restricted to severe manifestations of the disease; current evidence suggests that endothelial involvement is also common in mild or less severe cases [29,30]. Consequently, endothelial damage may be a consistent feature across the clinical severity spectrum of COVID and appears to be a broader phenomenon than previously appreciated. In view of this, identifying molecular targets to treat or prevent endothelial dysfunction (ED) is critical, as COVID-19 is considered a vascular disease characterized by the disruption of endothelial function and exacerbated inflammation, which determines the multiorgan complications of the disease [31]. In fact, the complex nature of SARS-CoV-2 infection serves to illustrate the pivotal function of the endothelium in both health and disease [31].

**Figure 1:**
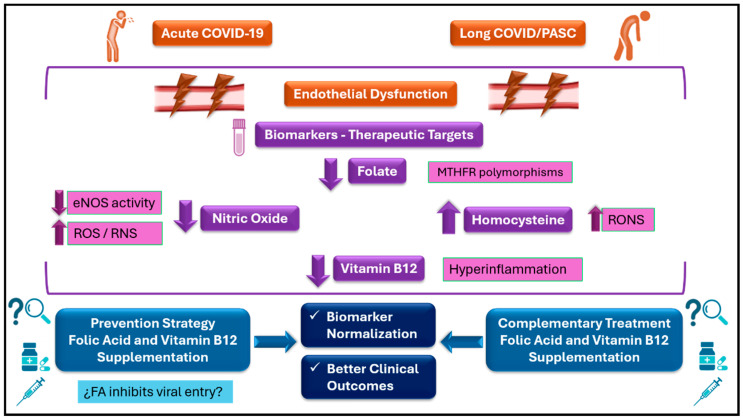
Conceptual overviewof the key findings discussed in this review. Endothelial dysfunction (ED) is present in both acute COVID-19 and Long COVID syndrome. The role of specific biomarkers dysregulated in ED, such as folate, homocysteine, nitric oxide and vitamin B12, is extensively discussed in this context. Additionally, folic acid and vitamin B12 supplementation is analyzed as a potential prevention strategy and complementary treatment to improve clinical outcomes and promote biomarker normalization. Created in BioRender. Massip Copiz, M. M. (2026) https://BioRender.com/300lyxj.

In the search for nutraceuticals capable of preventing ED, folic acid (FA) administration is proposed as a complementary therapy to alleviate symptoms and improve endothelial health. This review discusses the role of FA and related folate metabolism molecules, including homocysteine, in the ED associated with COVID-19. It further evaluates the potential benefits of FA supplementation for both the prevention and clinical management of the disease across its acute and chronic stages, as well as the role of vitamin B12 supplementation in long COVID syndrome. It is important to note that much of the current evidence remains largely emerging and occasionally indirect, and therefore, further mechanistic research is vital to fully validate these pathways. In Figure 1, a conceptual illustration synthesizes the key findings and maps out the core thematic structure discussed in this review.

## 2. Methods

This manuscript was designed as a narrative review focused on the potential role of folic acid, homocysteine and vitamin B12 in the endothelial dysfunction associated with COVID-19 in both the acute and long-term phases of the disease. Literature searches were conducted using the PubMed database to identify relevant articles published in English from 2020 to 2026. They were selected based on thematic relevance to the scope of the review, while additional relevant publications were identified through manual screening of reference lists. To ensure the inclusion of high-quality articles, the selection was restricted to those published in peer-reviewed journals. Search terms included combinations of the following keywords: “SARS-CoV-2”, “COVID-19”, “folic acid”, “folate”, “homocysteine”, “vitamin B12”, “endothelial dysfunction”, “gut microbiota” and related terms. Inclusion criteria comprised original experimental studies, clinical investigations, translational studies, observational studies, case series, major surveillance/statistical reports, systematic reviews, meta-analyses, and high-quality narrative reviews. Exclusion criteria included non-English publications, conference abstracts, and duplicate reports. Articles were identified through title, abstract, and full-text evaluation and were selected according to their relevance to the topics addressed in this review. Because the manuscript was designed as a narrative review rather than a systematic review, no formal meta-analysis was performed, and a structured risk of bias assessment was not applicable. Across database searches and manual reference screening, a total of 323 sources were selected and cited in the manuscript reference list.

## 3. Folate

### 3.1. Metabolic Functions of Folate

Folate, also known as vitamin B9, is a water-soluble vitamin that acts as a cofactor for different metabolic pathways within the cell. In its synthetic form is called folic acid and it is widely used for preventing neural tube defects during pregnancy and for treating megaloblastic anemia caused by folate deficiency [32,33]. As it cannot be synthesized by our body, folate must be obtained from diverse vegetables and animal sources such as green vegetables, citrus, legumes, nuts, liver and milk [34].

Folate is an indispensable micronutrient that functions as a critical cofactor in one-carbon metabolism, orchestrating essential biological functions such as nucleic acid (DNA and RNA) biosynthesis, amino acid metabolism, and methylation reactions [35].

Folate, together with other vitamins and trace elements, has an important role in supporting the innate and adaptive immune systems [36]. In addition to vitamin B12 and B6, folate participates in the regulation of intestinal immunity by promoting the survival of regulatory T cells in the small intestine [37,38]. It also maintains or enhances natural killer cell cytotoxic activity [39,40] and supports the Th1-mediated immune response [41]. Furthermore, as folate participates in the production and metabolism of antibodies, it facilitates an adequate humoral response to antigens [39]. Consequently, deficiency in this micronutrient negatively affects immune function and can increase the risk of acute infections [42].

Extensive in vitro and in vivo evidence demonstrates that folate and folic acid (FA) supplementation exerts multi-systemic therapeutic benefits [43]. These include cardiovascular, hematopoietic, and neural tube protection, as well as neuroprotective, antineoplastic, antihypertensive, and immunomodulatory effects. Mechanistically, these outcomes are primarily mediated through the regulation of homocysteine (Hcy) levels, oxidative stress, and inflammatory pathways [43]. Furthermore, folate modulates gut microbiota composition, thereby maintaining intestinal homeostasis and enhancing digestive health. Emerging evidence also indicates that folate mitigates depression by elevating monoamine neurotransmitter concentrations; however, the precise mechanisms linking folate to the gut microbiota–brain axis remain to be fully elucidated [43].

### 3.2. Folate and Gut Microbiota

While dietary ingestion is traditionally viewed as the primary mechanism for maintaining folate homeostasis, the human gastrointestinal tract serves as an autonomous site of vitamin B9 metabolism [44]. The gut microbiota and the host engage in a complex, bidirectional relationship regarding folate utilization [45]. While specific commensal taxa express the complete enzymatic machinery necessary for de novo folate biosynthesis, other auxotrophic organisms lack these genomic pathways and depend on cross-feeding mechanisms to satisfy their metabolic needs [45]. Dietary folates generally occur as polyglutamates, which require enzymatic cleavage to monoglutamates by glutamate carboxypeptidase II within the jejunal brush border before cellular uptake via the proton-coupled folate transporter (PCFT) [35]. However, structural evidence indicates that the human colon also displays high expressions of PCFT and various folate hydrolases [35]. This local anatomical architecture allows the host to absorb folates directly within the large intestine, capitalizing on the metabolic output of the resident colonic microbiota [35].

Within the microbial ecosystem of the gut, certain beneficial bacteria classified as probiotics play a vital role in executing de novo folate synthesis [46]. Genomic and physiological investigations highlight the genera *Lactobacillus* and *Bifidobacterium* as key modulators of this intra-luminal folate pool [46]. Genomic sequencing has revealed that the capacity to synthesize folate is highly strain-specific and not uniformly distributed across all lactic acid bacteria [46]. For instance, most wild-type *Lactobacillus* strains are auxotrophic for vitamin B9, meaning they actively deplete available folate to support their own cellular growth [46]. However, notable exceptions exist; *Lactobacillus plantarum* and *Lactobacillus reuteri* possess the complete biosynthetic pathways required to generate folate when supplied with the essential precursor para-aminobenzoic acid (pABA) [44,46].

In contrast, the genus *Bifidobacterium* demonstrates a widespread metabolic capacity for folate production [46]. Species such as *Bifidobacterium bifidum*, *Bifidobacterium longum*, and *Bifidobacterium pseudocatenulatum* regularly exhibit substantial de novo synthesis of the vitamin [46]. In vivo animal models have shown that administering these folate-producing probiotic strains significantly raises plasma folate levels, establishing that microbially produced vitamin B9 is successfully translated into systemic circulation [46]. Furthermore, human clinical evaluations have confirmed that target probiotic supplementation substantially boosts fecal folate concentrations, highlighting their potential as therapeutic tools to prevent localized deficiencies associated with mucosal inflammation and colonic malignancy [46].

The folate molecules synthesized by the enteric microbiota are highly bioavailable and easily integrated into the physiological pathways of the human host [47]. Once absorbed, this bioavailable microbial folate drives the folate-dependent one-carbon metabolic network, which is essential for preserving genomic stability and maintaining central nervous system health [48]. By supplying the necessary methyl groups, bacterial folate supports nucleotide biosynthesis (specifically purines and thymine), facilitating flawless DNA replication and repair mechanisms that mitigate the risks of chromosome instability and oncogenesis [48,49].

From a neurological standpoint, this microbially produced folate plays a vital protective role across the gut–brain axis [45,48]. It acts as an essential component in the remethylation of homocysteine to methionine [49]. Preventing an accumulation of systemic homocysteine is critical, as elevated levels lead to neurotoxicity, cognitive decline, and accelerated neurodegenerative pathologies like Alzheimer’s and Parkinson’s diseases [48]. Concurrently, the downstream synthesis of S-adenosylmethionine (SAM) drives the methylation of key neurotransmitters and myelin proteins required for stable cognitive function [48,49].

Finally, microbially synthesized folate acts as a crucial local homeostatic regulator within the intestine [45]. By participating in cross-feeding networks, prototrophic probiotic strains share synthesized folates with auxotrophic commensal taxa [45]. This cooperative interaction supports overall gut microbiota balance, maintains epithelial barrier integrity, and prevents the dysregulation of immune responses linked to inflammatory bowel conditions [45,46].

### 3.3. Gut Microbiota and COVID-19

Beyond its well-characterized interaction with mammalian respiratory epithelium, emerging evidence indicates that SARS-CoV-2 exhibits intricate interactions with the human gastrointestinal microbiome. Traditional models focus heavily on human angiotensin-converting enzyme 2 (ACE2) receptor-mediated enterocyte invasion; however, novel paradigms suggest a more complex, dual-nature biology. Specifically, research has demonstrated that SARS-CoV-2 replicates within human gut commensal bacteria and induces a classic bacteriophage-like replication cycle [50]. Utilizing advanced methodologies, investigators confirmed that the viral genome can be transcribed and translated directly within bacterial cells [50]. This process culminates in the lysis of beneficial gut commensals, introducing a distinct mechanistic layer to the development of gastrointestinal dysbiosis observed in COVID-19 patients [51]. Over time, this autonomous, extra-corporal replication allows the viral RNA load to steadily increase within fecal matrices independent of active human host tissue involvement [51].

The capacity of SARS-CoV-2 to infect and utilize bacterial hosts serves as a primary driver for its long-term persistence in the human gut. This continuous viral replication inside the microbial reservoir creates a perpetual state of local dysbiosis, significantly depressing the populations of symbiotic bacteria [50].

Because the human gut microbiota plays an indispensable role in synthesizing essential micronutrients, this targeted destruction creates profound metabolic repercussions. Commensal microflorae are responsible for the de novo synthesis of critical B-complex vitamins (such as folate, cobalamin, and pyridoxine) and vitamin K. Given the continuous viral lysis of these specific beneficial bacteria, severe vitamin deficiency becomes an eventual physiological certainty. The persistence of the virus within the intestinal lumen guarantees that the localized depletion of micronutrient-producing flora cannot readily recover, driving systemic nutritional deficits that further impair host metabolic homeostasis and compromise overall immune resilience [51].

Notably, dietary interventions can exacerbate or mitigate viral infection via the lung–gut axis, a critical pathway characterized by high levels of ACE2 receptor expressed within the gastrointestinal tract [52]. Thus, dietary bioactives play a promising role in protecting endothelial cells within the gastrointestinal tract, while bioavailable fractions can migrate to protect systemic vasculature in distant organs [52].

In addition to causing physical structural lysis and nutrient depletion, the interaction between SARS-CoV-2 and the gut microbiome alters bacterial translational pathways, triggering the synthesis of harmful xenobiotics. Investigators have demonstrated that this symbiotic disruption prompts the production of complex, toxin-like peptides resulting directly from the cross-kingdom interaction between the virus and bacteria [53].

Once secreted into the intestinal milieu, these highly bioactive toxins breach the mucosal barrier and gain entry into the host’s circulatory system [53]. Systemic dissemination of these microbially derived venom-like peptides exerts severe pathological actions on both the vascular endothelial lining and the human coagulation cascade [54]. The toxins degrade endothelial tight junctions, promote hyperpermeability, and stimulate intense localized thromboinflammation. Simultaneously, they disrupt the balance of the coagulation system, accelerating microvascular thrombosis and contributing to the multi-organ endothelial damage frequently encountered in severe and long-term COVID-19 syndromes [53].

## 4. Interactions Between Folic Acid and Homocysteine Metabolism

One-carbon metabolism comprises an intricate, compartmentalized network of biochemical pathways spanning the cytoplasm and mitochondria, serving as the metabolic hub for transferring single-carbon units essential for cell survival [55]. This metabolic machinery is structurally divided into two tightly synchronized components: the folate cycle and the methionine cycle [55]. Efficient homeostatic signaling between these two distinct pathways dictates whether single-carbon units are directed toward nucleic acid replication or utilized to maintain cellular methylation potential [33].

The physiological interface connecting these two metabolic axes governs the systemic balance between folic acid (vitamin B9) derivatives and the non-proteinogenic, sulfur-containing amino acid homocysteine [56]. Under standard conditions, a continuous influx of nutritional precursors and B-vitamin cofactors—specifically folic acid, cobalamin (vitamin B12), and pyridoxine (vitamin B6)—is required to keep this metabolic wheel turning [56]. Disruptions in this collaborative network impair cell signaling, halt nucleotide production, and trigger toxic metabolic accumulation [57].

**Figure 2:**
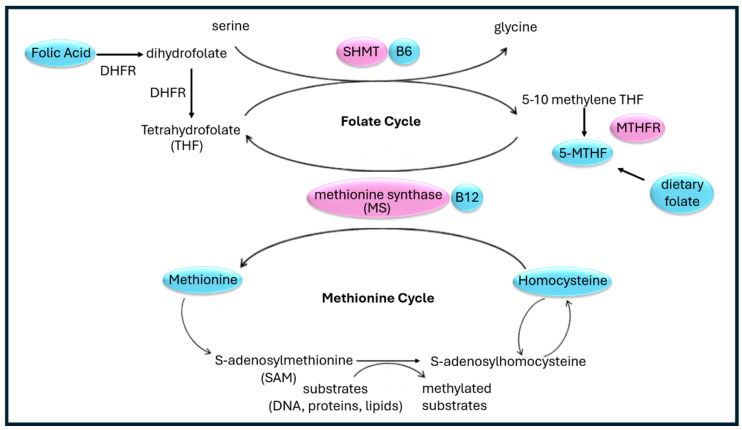
Interactions between folic acid and homocysteine metabolism. Abbreviations: 5-MTHF, 5-methyl-tetrahydrofolate; MTHFR, 5,10-methylene tetrahydrofolate reductase; DHFR, dihydrofolate reductase; THF, tetrahydrofolate; SHMT, serine hydroxymethyltransferase.

Beyond its role in the immune system, folate participates in the metabolism of homocysteine (Hcy) in conjunction with vitamin B12 and B6 [55]. Hcy is a key intermediate in the synthesis of amino acids methionine and cysteine and the universal methyl donor S-adenosyl-methionine (SAM), which participates in the biosynthesis of polyamines and methylation of numerous cell molecules [58] (Figure 2).

The crosstalk between FA and Hcy metabolic pathways is evidenced in the modulation of biochemical parameters across various pathologies. For example, total Hcy concentration often serves as a sensitive indicator of folate status, offering a valuable complement to plasma folate levels [59]. In particular, it was reported that plasma homocysteine levels are inversely correlated with serum concentrations of folate, vitamin B12, and vitamin B6 [60,61,62]. In fact, folate deficiency leads to an abnormal accumulation of homocysteine [63], achieving clinical significance at concentrations above 15 micromol/L (hyperhomocysteinemia). This threshold has been associated with an elevated risk of cardiovascular, cerebrovascular, and thromboembolic diseases [64].

It was demonstrated that both large and small vessels are susceptible to vascular injury when plasma homocysteine levels are high [65,66]. For example, in atherosclerosis, hyperhomocysteinemia (HHcy) causes endothelial injury by promoting inflammation and oxidative stress [64] and it plays an important role as an independent risk factor [67,68]. In the context of infectious diseases, such as COVID-19 and sepsis, elevated homocysteine levels may induce thrombotic events through substantial regulation of immunothrombosis pathways. Here, elevated Hcy levels have been shown to activate immune cells, including neutrophils, monocytes, platelets and endothelial cells, through a variety of molecular mechanisms, resulting in a thrombotic cascade [69].

While folic acid supplementation has a positive effect by lowering homocysteine values [70,71], it can also improve endothelial function through an Hcy-independent pathway [72]. Specifically, it reduces superoxide production [73,74] and regulates the metabolism of nitric oxide (NO), a potent vasodilator which has antithrombotic, antiangiogenic, and anti-inflammatory properties [75]. As will be discussed later, the administration of high doses of folic acid can prevent NOS dysfunction and promote endothelial health.

Despite the availability of folic acid in dietary supplements and fortified foods, consumption levels remain inadequate across Europe [76] and several other regions, including high-income nations [77,78,79]. Therefore, increasing FA intake should be considered as part of a comprehensive health prevention strategy, given its role in the prevention of neural tube defects, the treatment of anemia and the improvement of endothelial dysfunction. Indeed, as ED is involved in the pathogenesis of both severe acute COVID-19 and post-COVID syndrome, FA supplementation may potentially enhance patient recovery.

## 5. Endothelial Dysfunction in COVID-19

The vascular endothelium constitutes the innermost layer of blood vessels and its dynamic structure is vital to the maintenance of vascular health and homeostasis [80]. The term “endothelial dysfunction” describes an imbalance between vasodilator and vasoconstrictor factors produced by the endothelium, including a reduction in the bioavailability of nitric oxide (NO) [81]. This event leads to a disruption in the regulation of vasomotor tone and a shift towards a pro-inflammatory state in the endothelium [82,83].

Current evidence suggests that COVID-19 is a pan-vascular disease, highlighting the endothelium as its “Achilles heel” [84,85,86]. In fact, while SARS-CoV-2 infection mainly impacts the respiratory tract, COVID-19 can manifest as a multi-organ disease due to endothelial dysfunction, endotheliopathy and endotheliitis. These conditions involve a shift in the vascular equilibrium towards vasoconstriction, and a pro-inflammatory and pro-coagulant state [87,88,89]. In COVID-19, diverse factors contribute to the development of ED, such as “the cytokine storm”, generating a hyperinflammatory state [90], renin–angiotensin–aldosterone system imbalance [91] and thrombotic microangiopathy (immunothrombosis) [91].

Beyond this characteristic hyperinflammatory response, the vascular endothelium serves as a direct target for SARS-CoV-2 via ACE2 (angiotensin-converting enzyme 2) [1]. Other molecules expressed in endothelial cells also participate in viral mechanisms of fusion and entry, such as receptors (neuropilin-1 and neuropilin-2; NRP-1/NRP-2), proteases (TMPRSS2 and furin), gangliosides and glycoproteins (heparan sulfate and sialic acid-containing glycoproteins) [92]. Consequently, endothelial cell infections and subsequent endotheliitis (inflammation of the endothelium) have been reported to contribute to COVID pathology, particularly when the viral load is high [93,94]. Nevertheless, endothelial cell damage results primarily from complement activation and membrane attack complex rather than direct viral cytopathic effects [95].

As previously mentioned, vascular impairment is principally attributed to the indirect consequences of endothelial activation, resulting from a systemic inflammatory response characterized by an excess of cytokines [93]. The activation of these mechanisms triggers a state of hypercoagulability and inflammation in the endothelium, which results in increased vascular permeability, thrombosis, vasoconstriction, production of reactive oxygen species, and the induction of apoptosis or pyroptosis [96]. This endothelial inflammation induced by SARS-CoV-2 favors a systemic impairment of microcirculatory function in multiple vascular beds, and the subsequent clinical sequelae observed in patients with acute COVID-19 [93].

The sequence of ED events begins with the cytokine-induced impairment of the endothelial barrier function, leading to basement membrane breakdown, endothelial cell death, and a reduction in vascular integrity [83]. Principally, the interactions between two viral proteins and cellular receptors are involved in the perpetuation of this inflammatory process within the cells and tissues. Initially, the virus S-protein binds to ACE2, thereby activating the type 1 angiotensin II receptor (AT1R) and leading to the overactivation of NADPH-oxidase 2 (Nox 2), which is a major producer of cellular ROS (reactive oxygen species) [97]. Secondly, the nucleocapsid binds to the NOD-like receptor protein 3 (NLRP3) inflammasome within the host cells, and this interaction consequently leads to pore formation in cell membranes and the release of cytosolic components, resulting in cell death [98].

In fact, the NLRP3 inflammasome has been identified as a key factor in the induction and perpetuation of the cytokine storm syndrome (CSS), a condition characterized by excessive inflammation resulting from uncontrolled production of cytokines. This unregulated production of cytokines has been linked to significant systemic damage, affecting vital organs such as the lungs, brain, heart, and kidneys [89]. Indeed, the CSS has been associated with endothelial impairment within the microvasculature and is concomitant with systemic dysregulation of the fibrinolysis-coagulation balance [89].

Similarly, the generation of abnormally elevated levels of ROS in the context of a COVID-induced cytokine storm leads to the irreversible oxidation of a diverse range of macromolecules, ultimately resulting in cellular injury and subsequent impairment of organ function [99]. Clinical investigations underscore the pivotal role of oxidative stress in triggering endothelial damage, thereby increasing the risk of adverse outcomes in patients with acute and long COVID-19 [99]. Here, oxidative stress is a consequence of increased ROS generation and diminished NOS bioavailability. Both factors contribute to endothelial dysfunction, which manifests through upregulated adhesion molecule expression, enhanced vascular permeability, platelet adhesion and aggregation, proliferation of smooth muscle cells, and vasoconstriction [80]. Moreover, reduced NO production by the injured endothelium may enhance platelet activation and clotting induction [100] via the clotting cascade, ultimately promoting thrombus formation [27].

Furthermore, direct interaction between platelets and SARS-CoV-2 also increases the risk of thrombosis. When virions are internalized, a rapid digestion occurs that leads to programmed cell death (necroptosis and apoptosis), and the subsequent release of extracellular vesicles [101]. This rapid reaction may result in immune system dysfunction and the onset of thrombosis [101]. Therefore, these mechanisms, together with a state of immune dysregulation and in conjunction with ongoing viral reservoirs, have been shown to cause a chronic inflammatory response leading to endothelial damage [102].

Following severe endothelial injury, three distinctive angiocentric features were primarily observed in the lungs of patients with COVID-19 [103]: the disruption of the endothelial cell membranes, vascular thrombosis with microangiopathy and occlusion of the alveolar capillaries, and the development of unexpected new vessels [103]. Accordingly, research indicates that this chronic inflammation state was not limited to the lungs, and it clearly dysregulates the homeostatic balance between procoagulant and anticoagulant factors in the vascular endothelium, which results in arterial and venous thrombosis [104]. Indeed, patients with severe COVID-19 frequently experienced pulmonary and systemic vascular complications, which may manifest as pulmonary embolism, deep vein thrombosis, and major cardiovascular (CV) events [104].

Endothelial dysfunction represents a significant contributing factor to the poor progression of SARS-CoV-2 infection and the subsequent adverse clinical outcomes [105]. Post-mortem examinations have revealed endotheliitis in 90% of cases and thrombosis in 30–60% of small- and medium-sized vessels [106]. Furthermore, studies employing nailfold video capillaroscopy (NVC) to assess microcirculatory alterations in response to acute infection have demonstrated an increment in microthrombus formation and a markedly slow or discontinuous flow velocity during the acute phase [107]. Conversely, long-term observations indicate a reduction in capillary number [108] accompanied by capillary dilation.

Additionally, the vascular consequences of SARS-CoV-2 infection are exacerbated by the endothelial dysfunction already established in patients with chronic diseases, such as cardiovascular disease (CVD) [109], cardiometabolic disorders [110], diabetes mellitus [111], hypertension [112,113] and obesity [114]. In fact, ED is associated with cardiovascular complications, especially in patients with metabolic disorders [115]. In some cases, baseline vascular damage has already been induced by metabolic syndrome, glucotoxicity, or lipotoxicity from diets high in reducing sugars and saturated fats [52]. All these comorbidities are generally related to a severe clinical course of COVID-19, requiring hospitalization or intensive care unit (ICU) admission [116,117].

Indeed, one of the lethal consequences in severely ill patients is a procoagulant and antifibrinolytic state induced by chronic endothelial dysfunction [118]. Fundamentally, in the lung microenvironment, ED is a primary driver of respiratory failure, alongside the formation of microthrombi [119]. Here, the impaired vasoconstriction—which results in significant shunting—along with thrombotic microangiopathy [120,121,122], explains why these patients often maintain relatively good lung compliance despite poor oxygenation [123].

In summary, ED related to COVID-19 may lead to macro- and microvascular thrombotic events, with subsequent impairment of organ perfusion. Undoubtedly, the implementation of therapeutic strategies aimed at preventing or mitigating endothelial dysfunction has the potential to markedly enhance prognosis and reduce mortality rate of COVID-19 [105].

## 6. Nitric Oxide and Folate Metabolism in COVID-19

As detailed in Section 5, one of the key factors of endothelial dysfunction is the reduced nitric oxide synthesis and bioavailability. This process stems from dysfunctional endothelial nitric oxide synthase (eNOS), which limits local NO production.

**Figure 3:**
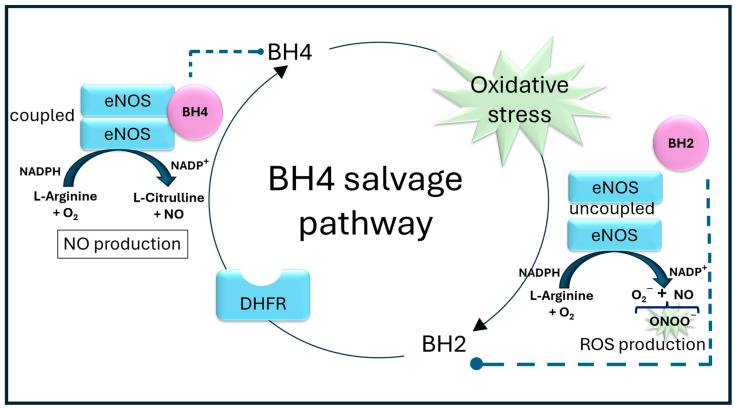
Endothelial nitric oxide synthase (eNOS) function and BH4 salvage pathway. The folate-dependent enzyme dihydrofolate reductase (DHFR) participates in the recycling of BH4 co-factor, which contributes to the stabilization of eNOS and the efficient production of NO.

In normal conditions, folate promotes NO production by increasing eNOS activity via its primary metabolite, 5-Methyl tetrahydrofolate (5-MTHF). 5-MTHF contributes to potentiate tetrahydrobiopterin (BH4) levels, a cofactor of the eNOS enzyme [124,125], and it also exerts antioxidant effects via free radical scavenging, promoting eNOS coupling and maintaining its enzymatic function [126,127]. These antioxidant properties are critical because elevated production of ROS (reactive oxygen species) reduce BH4 bioavailability, which undergoes rapid oxidation to 7,8-dihydrobiopterin (BH2) within the circulatory system [82,128] (Figure 3). Thus, in the absence of BH4, uncoupling of eNOS occurs, resulting in the production of superoxide radicals instead of nitric oxide [128] and exacerbating oxidative stress.

As mentioned above, the relevance of BH4 lies in its role in maintaining the dimeric structure of eNOS [129,130,131]. At physiological levels, BH4 stabilizes the eNOS dimer, enabling the efficient production of NO. Conversely, insufficient BH4 availability has been shown to induce a shift in eNOS towards a monomeric state. This transition results in the uncoupling of eNOS and the subsequent production of superoxide rather than NO, further exacerbating the oxidative disruption of the dimeric eNOS complex [132] (Figure 3). In endothelial cells, BH4 levels can be regulated via the “salvage” pathway, in which the folate-dependent enzyme dihydrofolate reductase (DHFR) catalyzes the reduction of BH2, thereby facilitating the restoration of BH4 levels [133].

As shown in Figure 3, DHFR plays a pivotal role in the reduction of BH2 back to BH4 via the BH4 salvage pathway [134,135]. This pathway is significant in maintaining an optimal BH4:BH2 ratio, thereby supporting NO production. In vitro studies demonstrate that inhibiting DHFR reduces BH4 and increases BH2 levels, leading to the enzymatic uncoupling of eNOS and elevated production of ROS [134,135]. Furthermore, superoxide anions and oxygenated free radicals facilitate the oxidation of BH4 to its inactive form (BH2) [136,137], contributing to the perpetuation of BH4 deficiency. Consequently, as BH2 competes with BH4 for the eNOS binding site (Figure 3), determining the BH4/BH2 ratio is a critical factor in eNOS dimerization and function [138,139]. Therefore, the oxidation of BH4 serves as both a cause and a consequence of monomerization and subsequent uncoupling of eNOS. This dynamic process creates a “vicious cycle” that escalates oxidative stress within the cell [140].

BH4 is clinically significant due to its essential role in diverse physiological processes, including self-protection against NO toxicity, vascularization, inflammation, and oxidative status [140]. As a result, low bioavailability of substrates or cofactors, or high levels of oxidative stress, impair the ability of the endothelium to produce normal levels of NO, leading to endothelial dysfunction [75]. Thus, a complementary approach to treating ED could involve enhancement of BH4 synthesis or mitigation of its oxidative degradation [140]. For instance, the administration of folic acid, and specifically its active metabolite 5-methyltetrahydrofolate, can reduce BH4 oxidation while accelerating the rate of BH2 recycling to BH4 via enhanced DHFR activation. This dual mechanism ultimately maintains BH4 levels within the physiological norm [124,141,142,143]. Additionally, in conditions of acidosis or hypoxemia, NO can be restored independently of eNOS through dietary inorganic nitrate, which can be converted to NO [144].

As observed, various mechanisms regulated by folic acid contribute to preventing endothelial dysfunction and promoting endothelial health, thereby exerting beneficial effects during SARS-CoV-2 infection. This is particularly significant given the reduced levels and bioavailability of endothelial nitric oxide found in COVID-19 patients [145]. Hence, treating ED may improve prognosis in both acute and long-term phases of COVID-19, as SARS-CoV-2 substantially affects the vascular system [146].

Moreover, folic acid was shown to improve cardiovascular health by inducing BH4-dependent eNOS activation. Consequently, folic acid supplementation may represent a viable strategy for preventing eNOS uncoupling in cardiovascular complications [147]. It is worth noting, however, that while the mechanistic rationale for folate in supporting eNOS coupling is robust, clinical evidence in human trials remains mixed. Specifically, studies evaluating FA in cardiovascular cohorts have yielded variable results, underscoring the need for more robust clinical proof [148].

Finally, to potentiate the efficacy of FA, it is essential to investigate additional molecular mediators involved in the signaling pathways that could prevent ED and enhance NO bioavailability. Principally, the balance between relaxing and contracting factors, homocysteine levels and the modulation of the ratio between reactive oxygen species and scavenger molecules warrants particular attention.

## 7. Folate and Homocysteine as Biomarkers in COVID-19

Since the beginning of the pandemic, diverse studies have identified alterations in folate and homocysteine levels among patients with COVID-19. Notably, in 2020, Itelman and colleagues first reported decreased serum folic acid levels in severely ill individuals in Israel [149]. Furthermore, a distinct relationship between FA metabolism and susceptibility to SARS-CoV-2 infection was characterized in Italy and Spain—two countries significantly impacted at the onset of the pandemic. These populations exhibit a genetic predisposition to the single nucleotide polymorphism (SNP) C677T in the MTHFR (5-10-methylenetetrahydrofolate reductase) gene, responsible for a key enzyme in folate metabolism (Figure 2). This SNP reduces the MTHFR activity, resulting in higher levels of Hcy and lower levels of folate [150,151]. In fact, the suboptimal folate status observed in several European countries positively correlated with COVID-19 incidence, severity and mortality [152,153]. This association has been shown to be even more accentuated in Latino populations compared to global averages [154].

Recent clinical evidence has highlighted the critical role of MTHFR mutations in COVID-19 severity, particularly within the context of hereditary thrombophilia. Heterozygous variants, specifically C667T and A1298C, have been implicated in the etiology of hereditary thrombophilias. It was reported that these mutations are associated with a significantly elevated risk of thrombotic complications during SARS-CoV-2 infection, contributing to increased mortality rates of COVID-19 patients [155].

Supporting these findings, a prospective cross-sectional study revealed a higher prevalence of MTHFR heteromutation among both pediatric and adult cohorts presenting with severe COVID-19 [156]. The study further established that the heterozygous genotypes of C677T and A1298C serve as independent risk factors for thrombosis in critical cases [156]. Therefore, MTHFR polymorphisms, alongside homocysteine levels, are now recognized as clinical modulators of COVID-19 incidence and severity. The epidemiological distribution of the MTHFR C677T variant appears to correlate closely with regional mortality trends, specifically in China and Italy, where the combined prevalence of CT and TT genotypes reaches 67.1% and 66.3%, respectively [157,158]. These high frequencies may provide a genetic explanation for the disproportionately elevated COVID-19 mortality rates observed in these populations [156].

Subsequently, various investigations have examined relationships between folate levels and clinical outcomes in patients with COVID-19. Voelkle and colleagues reported that elevated FA levels were associated with a reduced risk of severe disease [159]. Specifically, individuals with a mild clinical course exhibited median admission FA values over 1.5-fold higher than those with severe cases [159]. The authors highlighted the contribution of micronutrient deficiencies, including low FA levels, to adverse COVID-19 outcomes [159]. Nevertheless, they noted that the clinical utility of these findings might be limited, as observed levels were only marginally below established cut-off values [159]. Similarly, Meisel and colleagues documented decreased folate levels in COVID-19 patients, though these did not reach statistical significance regarding clinical prognosis [160].

Conversely, a retrospective observational study proposed folate as a biomarker for poor prognosis in COVID-19. In this cohort, folate levels were significantly lower in patients who required ICU hospitalization and intubation compared to those who did not, as well as in those with fatal outcomes compared to those who were discharged [161]. Although mean folate levels remained within the reference range, these findings underscore the potential importance of monitoring folate status during COVID-19 management [161].

Furthermore, studies have reported that plasma folate levels are significantly reduced in patients with both severe and mild COVID-19 relative to healthy controls [162]. This observation is particularly relevant when examining specific clinical subgroups. For instance, the prevalence of folate deficiency in people with obesity [163] may contribute to their increased susceptibility to COVID-19 [114]. Data regarding the pediatric population remain limited; however, folate deficiency has been documented in 15% of hospitalized pediatric cases [164]. More recently, a case–control study by Paduano and colleagues has shown a significant reduction in serum folate concentrations among COVID-19 patients [165], reinforcing the importance of folate intake for prevention.

In addition to FA metabolism, homocysteine metabolism has been implicated in the pathophysiology of COVID-19. Specifically, increased Hcy levels have been identified in patients with mild disease, acting as a predictive marker of subsequent imaging progression observed on chest CT (computed tomography) in COVID-19 individuals [166].

Similar elevations in Hcy have been reported in other viral infections [167,168,169], such as human papillomavirus (HPV), hepatitis C virus (HCV) and human immunodeficiency virus (HIV), suggesting its broader relevance as a clinical biomarker and its significance in the context of COVID-19. For instance, patients with HIV exhibit a correlation between high Hcy and low folate levels, consistent with observations in COVID-19 [168]. In subjects with HPV, folate levels are also decreased, though this decline is presumably associated with an insufficient cellular immunity [167]. Consequently, Hcy is a promising biomarker for predicting clinical outcomes in hospitalized COVID-19 patients [170]. Its application could facilitate risk stratification to improve clinical management, monitor serious complications [171] and guide the timely administration of anticoagulant or fibrinolytic therapies [172].

Other studies have consistently underscored the clinical utility of Hcy as a biomarker for COVID-19-related hospitalizations. Elevated Hcy levels upon emergency department admission serve as a viable indicator for intensive care requirements and a predictor of poor prognosis in cases of COVID-19-associated pneumonia [173]. Notably, clinicians found that each unit increase in Hcy doubled the risk of mortality in patients with COVID-19 [173]. This observation is reinforced by further studies identifying significantly higher serum Hcy levels in severe COVID-19 cases [174]. Similarly, in pediatric cohorts, high serum total homocysteine levels correlate with COVID-19 severity, radiological chest findings and laboratory inflammatory parameters, such as D-dimer, ferritin, alanine transaminase (ALT), aspartate transaminase (AST) and blood urea nitrogen (BUN) [175].

Currently, the clinical relevance of Hcy is more prominent in pathologies characterized by ED and vascular damage, as seen in COVID-19. Here, elevated Hcy levels exert deleterious effects on the vascular endothelium, triggering platelet activation and thrombus formation, which culminates in a hypercoagulable state [176]. For instance, a case–control study demonstrated that serum Hcy levels exceeding 13.7 μmol/L are associated with an increased risk of COVID-19 and a predisposition to coagulation [177]. It is hypothesized that homocysteine modulates vascular endothelial function through several mechanisms, including induction of oxidative stress, reduction in nitric oxide bioavailability, stimulation of smooth cell proliferation, and alteration of the elastic wall properties. Furthermore, the oxidative inactivation of nitric oxide may contribute to the development of HHcy in ED [176].

While larger studies are necessary to confirm serum Hcy as a biomarker for severe COVID-19, it also represents a significant clinical target that should be stabilized through FA and vitamin B12 supplementation. This metabolic context is further complicated by reduced levels of folate and various B-vitamins, which contribute to hyperhomocysteinemia across numerous pathologies. Notably, this is particularly evident in CVD and central nervous system disorders [178] that share clinical features with both the acute and long-term phases of COVID-19.

Finally, in contrast to the aforementioned results, several studies have indicated an inconsistent relationship between Hcy levels and COVID-19 severity [179], often finding no significant variations in folate, vitamin B12 and Hcy markers. For instance, a South Korean study involving 50 hospitalized patients with COVID-19 observed no marked deficiencies in vitamin B12 and folate [180]. Other authors provide scant evidence to support possible associations between micronutrient concentrations, including folate and vitamin B12, and COVID-19 outcomes, though they are based only on genetically predicted circulating micronutrient concentrations [181]. Prior investigations indicate that folate and Hcy levels did not significantly differ across cohorts, despite paradoxically elevated vitamin B12 levels in severe cases [182]. Conversely, an independent study found no such disparity in vitamin B12 levels between pediatric patients with COVID-19 and healthy controls [183].

Ultimately, more extensive research is required to establish a definitive conclusion given the limitations of these studies, including the limited sample sizes, heterogeneous preexisting comorbidities, and varied clinical severities across COVID-19 subjects.

## 8. Folic Acid Administration in Acute COVID-19

The therapeutic potential of FA for the prevention of SARS-CoV-2 infection or as a complementary treatment for acute COVID-19 has been a subject of discussion since the outbreak of the pandemic. Numerous studies have underscored the role of FA in regulating homocysteine metabolism and addressing endothelial dysfunction during the acute phase of the disease.

Some authors proposed that SARS-CoV-2 infection aggravates the cellular metabolism of the homocysteine pathway, and folic acid, together with vitamin B6 and B12, has been suggested to suppress complications [184], particularly those associated with pulmonary hypertension [185] and hyperhomocysteinemic hypercoagulability [186]. Other investigators have indicated that in populations characterized by poor dietary habits and/or high prevalence of MTHFR 677T alleles, targeted supplementation with FA and B-vitamins could be able to lower Hcy levels, potentially reducing COVID-19 infection rates and overall mortality [154].

In this context, increasing FA intake is also recommended as a preventive measure against comorbidities that exacerbate COVID-19 risk. As previously noted, FA prevents or reverses cardiovascular disease progression by increasing NO bioavailability [75]. In fact, evidence suggests that additional folic acid supplementation (≥5 mg/day) serves as an effective strategy for improving cardiovascular health [75]. Other publications associate the CV benefits of folic acid supplementation with its ability to lower homocysteine levels [187,188], but this mechanism remains a subject of debate.

Furthermore, FA plays a significant role in stroke prevention among patients with CVD [189] and is associated with reduced stroke mortality across the general population [190]. Additionally, in patients with coronary artery disease (CAD), the combination of folic acid and antioxidant vitamins lowers the risk of endothelial dysfunction [191,192]. Supporting this, a randomized controlled trial demonstrated that high doses of FA (5 mg/day) administered for over four weeks improved flow-mediated dilation (FMD) and lowered plasma homocysteine concentration in patients with CAD [193,194]. These findings further justify high-dose folic acid supplementation to enhance endothelial function.

Similar observations have been reported in patients with hypertension, where high-dose FA supplementation (≥5 mg/day) positively influenced systolic blood pressure reduction [195]. Notably, doses exceeding 5 mg/day of folic acid are necessary to improve NO-dependent endothelial vasodilation [196]. Consequently, the modest impact of folic acid observed in some studies is primarily attributable to the doses employed in those clinical trials [196,197]. It should be noted that the efficacy of FA in improving flow-mediated dilation (FMD) has been described in patients with comorbidities characterized by endothelial dysfunction, such as cardiovascular disease or diabetes [198,199,200], yet this effect was not observed in the context of chronic kidney disease [201,202]. Therefore, the presence of concomitant renal disease in COVID-19 patients is a critical factor to consider when developing FA therapeutic regimens.

Correspondingly, administration of the active form of FA, 5-MTHF, may be considered a potential complementary or preventive treatment for elderly populations susceptible to severe COVID-19 presentations. This is supported by reports that 5-MTHF increases NO-mediated vasodilation in healthy older adults (>60 years) [196] and decreases oxidative stress [203] in postmenopausal women. Based on this evidence, high-dose supplementation of folic acid or 5-MTHF has been proposed as a therapy for pulmonary hypertension in COVID-19 patients, specifically by modulating eNOS function and enhancing NO bioavailability [204].

Beyond its role as a complementary intervention against COVID-19-related systemic pathology, the impact of folate intake has been evaluated as a preventive measure against SARS-CoV-2 infection. Using a standardized protocol, a large population-based study investigated the associations between infection risk and dietary intakes (nutrients, food groups and overall diet quality). The resulting seroprevalence data revealed that higher folate intake correlated with a reduced likelihood of SARS-CoV-2 infection [205]. Correspondingly, another study observed that among healthy young non-obese individuals, those with SARS-CoV-2 had a lower dietary intake of folate than those without the virus [206]. These findings support the suggestion that pregnant women be supplemented with 400 mcg per day of FA to protect them against the risk of SARS-CoV-2 infection [207].

Several studies have elucidated potential mechanisms by which FA may directly inhibit SARS-CoV-2 infection. Notably, FA and its derivatives exhibit strong and stable binding affinity for various SARS-CoV-2-associated protein targets [208]. Virtual screening models indicate that FA could interact with both mutant and wild-type SARS-CoV-2 helicases, potentially impeding viral replication [209]. Furthermore, computational analysis by Pandya and colleagues demonstrated that FA could bind to several SARS-CoV-2 proteins, including spike, RdRp (RNA-dependent RNA polymerase), and NSP3 (non-structural protein 3) [210]. Moreover, it could interact with furin protease, a cellular protein involved in viral entry, exerting a robust inhibitory effect [210].

Research involving cell culture models has further characterized FA as a potential inhibitor of the SARS-CoV-2 nucleocapsid protein [211]. Subsequently, Pennisi and colleagues utilized pseudovirus technology to confirm that FA inhibits the entry of alpha and omicron SARS-CoV-2 variants [212]. Molecular modeling in their study identified specific FA binding sites within the RBD (receptor binding domain) of the spike protein in both variants [212].

More recently, Ojeda-Galván and colleagues found that the folic acid metabolite, 7,8-dihydrofolate (DHF), binds to ACE2, serving as a physical barrier that prevents RBD-receptor interaction [213]. Additionally, folic acid may inhibit the interaction between the S-glycoprotein and the endothelial cell receptor NRP-1, thereby preventing viral internalization [214]. In fact, as NRP-1 plays a pivotal role in cardiovascular pathophysiology, FA may offer therapeutic benefits for CV complications arising from COVID-19 [214]. In summary, current evidence suggests that FA exerts antiviral effects through direct interaction with key viral and host proteins, inhibiting critical stages of the viral life cycle such as entry and replication. These findings highlight the therapeutic potential of folic acid, warranting further investigation to fully elucidate its precise mechanism and clinical efficacy.

Beyond these immediate effects, FA plays a critical role in modulating SARS-CoV-2 receptors within host cells. Research indicates that folic acid treatment and 5-10-methylenetetrahydrofolate reductase (MTHFR) overexpression inhibit ACE2 expression by regulating promoter methylation [215]. Consequently, FA treatment reduces the binding affinity of the SARS-CoV-2 spike protein to host cells, thereby decreasing viral invasion and neutralizing antibody production in mice [215]. According to these findings, higher folic acid supplementation may downregulate ACE2 expression and reduce SARS-CoV-2 transmissibility, offering potential for infection control and prevention [215].

Finally, FA has demonstrated clinical efficacy in managing COVID-19. The administration of 1 g of FA, integrated into a multidrug therapeutic regimen, has been utilized for outpatients presenting with severe symptoms. In this cohort, hospitalization and mortality rates were markedly low [216]. Similar results were observed in a blinded, randomized controlled clinical trial in which hospitalized COVID-19 patients received a nutritional support system containing FA alongside vitamins, minerals, fiber, omega-3, amino acids, B complex and probiotics [217]. The intervention cohort (IC), which received 10 mg of FA per day for 21 days, demonstrated a significantly lower mortality rate than the control group (CG). Furthermore, compared to the CG, the IC showed a 10% reduction in progression to Mechanical Ventilation Assistance (MVA), a 15-day reduction in the intubation period, and a 38% increase in survival among intubated patients [217]. Nevertheless, further large-scale clinical trials are required to fully validate this therapeutic strategy.

Notably, despite the documented efficacy of adequate folate intake, excessive exposure is increasingly linked to deleterious health outcomes [218]. While these negative associations are widely recognized, their precise causal mechanisms have yet to be elucidated. Literature indicates diverse deleterious effects secondary to folate excess, including pregnancy complications, offspring disease risk, neurodevelopmental deficits, altered immune/allergic responses and accelerated carcinogenesis [218]. Consequently, although the tolerable upper intake level (UL) for folic acid is established at 1 mg/day for adults, defining the threshold for excessive folate intake remains elusive due to a lack of robust dose–response data and limited clinical documentation of adverse effects [219].

It is important to point out that, during all FA treatments, it is essential to monitor vitamin B12 levels, given its role in folate metabolism (Figure 2). In some cases, the increment of FA supplementation, with a possible vitamin B12 deficiency background, may mask the cognitive symptoms of pernicious anemia [220,221] (see Section 10). In these patients, high intakes of FA can correct the megaloblastic and macrocytic anemia typically caused by vitamin B12 deficiency by bypassing the B12-dependent methionine synthase reaction and directly supplying tetrahydrofolate for DNA synthesis [222]. While this hematological correction effectively masks the primary biomarker of vitamin B12 deficiency during routine blood counts, it fails to halt the underlying pathology. Consequently, neurological degeneration—such as subacute combined degeneration of the spinal cord—can progress completely undetected, often accelerating toward irreversible cognitive and motor impairment [223].

This clinical concern is further underscored by epidemiological evidence demonstrating that individuals with high serum folate levels paired with low vitamin B12 status exhibit a significantly elevated risk of cognitive decline and exacerbated anemia compared to those with balanced micronutrient levels [224]. Consequently, prolonged and unmonitored FA supplementation carries the distinct clinical risk of masking an underlying vitamin B12 deficiency, potentially delaying necessary intervention. Therefore, routine surveillance of vitamin B12 status is strongly recommended when initiating FA therapy to ensure patient safety and therapeutic efficacy. Clinically, this represents a significant concern in COVID-19, where vitamin B12 deficiency is proposed as both a relevant marker and a risk factor that can aggravate the severity of the disease in elderly patients or individuals with diabetes [225].

In contrast to the aforementioned studies, some authors have reported that SARS-CoV-2 hijacks host folate and one-carbon metabolism for viral replication [226]. This supports the hypothesis that lower folate levels in SARS-CoV-2-infected cells result from the high demand for ribonucleotide synthesis required for viral replication [226]. Consequently, it has been suggested that antifolates may exert both antiviral and anti-inflammatory effects in the context of COVID-19 treatments [226]. Notably, Stegmann et al. observed in cell culture models that the folate antagonist methotrexate diminishes SARS-CoV-2 replication and enhances the antiviral efficacy of remdesivir [227]. These findings are further supported by an observational case–control analysis from the UK Biobank, which indicated that individuals receiving FA supplements have a higher incidence of COVID-19 diagnosis and COVID-19-related mortality, whereas methotrexate may reduce this risk [228]. However, due to the several limitations of this observational study, a causal link between FA supplementation and COVID-19-related mortality cannot be definitively inferred [228]. Undoubtedly, larger-scale studies involving patients with mild and severe COVID-19 are needed to provide conclusive data.

## 9. Long COVID and the Consequences of Endothelial Dysfunction

Beyond the acute phase of COVID-19, some patients develop a chronic pathology termed “long COVID” (LC) or “post COVID-19 condition”, defined by the persistence of symptoms for at least 2 months [229]. This syndrome, also referred to as “post-acute sequelae of SARS-CoV-2 infection” (PASC), is primarily characterized by neurocognitive disorders, chronic fatigue, shortness of breath, cardiovascular and coagulation disorders and immune system dysregulations [229]. Cardinal manifestations include brain fog (or cognitive dysfunction) [230], general fatigue, dysautonomia (which commonly manifests as postural orthostatic tachycardia syndrome (POTS)) [231] and post-exertional malaise [19]. Additionally, several long COVID-associated complications, including heart disease, diabetes and myalgic encephalomyelitis (ME), are classified as lifelong chronic conditions [232,233].

**Figure 4:**
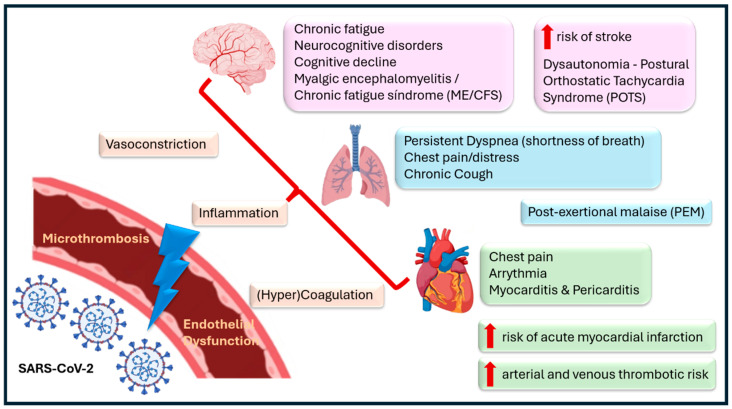
Principal symptoms of long COVID mediated by endothelial dysfunction. Created in BioRender. Massip Copiz, M. M. (2026) https://BioRender.com/ejvoosf.

A substantial number of studies indicate that three principal symptom groups are associated with long COVID: neurological–neuropsychiatric, pneumological and cardiovascular (Figure 4). The first group mainly comprises: the specific “COVID-19-brain fog” (characterized by attention deficit, impaired concentration, and reduced memory, information processing speed and executive function) and the autonomic nervous system involvement evidenced by the high prevalence of POTS [234,235,236].

Regarding the second group, the most prevalent respiratory symptom reported was dyspnea (shortness of breath), followed by post-activity polypnea (abnormal rapid breathing after exertion), chronic cough, chest distress, and chest pain, assessed across both children and young adults [237,238]. The structural alterations—such as pulmonary fibrosis and other patterns of lung damage—are proposed mechanisms for respiratory symptoms [239,240,241,242]. In turn, endothelial dysfunction may simultaneously contribute to reduced pulmonary functional indices [243] (Figure 4).

In the third group, cardiovascular complications (including myocarditis, pericarditis and an increased risk of acute myocardial infarction and thrombosis) significantly impact morbidity and mortality, consistent with trends in other viral respiratory infections. The instability of chronic CVD may be exacerbated during acute viral infection, potentially resulting in myocarditis and subsequent cardiac impairment. Furthermore, systemic inflammation and procoagulant effects can lead to coronary plaque rupture and thrombosis [244,245]. Hence, the established correlation between SARS-CoV-2 and stroke, combined with its associated risk of myocardial infarction, suggests a broader relationship between impaired blood flow and heightened CVD risk in this context [246,247,248] (Figure 4).

One of the aspects characterizing LC/PASC is that patients can suffer this chronic condition regardless of the initial severity of the acute phase of the disease [249,250]. Although the prevalence of long COVID is currently unknown, it has been estimated that one in three survivors may develop it [251,252]. This chronic syndrome impacts individuals across all demographic groups—including children [253], older adults [254], and diverse racial and ethnic backgrounds—irrespective of sex or gender [19,255]. Furthermore, this condition affects individuals with varying levels of baseline health status [19,255]. In pediatric population, a meaningful subset of children experiences symptoms that severely disrupt daily physical and cognitive functioning [256]. Notably, these LC symptoms are highly heterogeneous and characteristically fluctuate or relapse over time, making early diagnosis particularly challenging [256].

As a result, it is becoming evident that a new global health burden has emerged. This is characterized by the transition of patients with post-COVID (post-recovery from acute SARS-CoV-2 infection) to a virus-free disease state, accompanied by the persistence or development of chronic clinical manifestations [6]. Data indicates that 25–70% of individuals who survive COVID-19 and are free of SARS-CoV-2 infection continue to exhibit symptoms associated with viral-induced HMRD (human metabolic reprogramming and dysregulation). This encompasses a broad spectrum of presentations, ranging from persistent exacerbations of pre-existing conditions to the onset of novel pathologies [6].

Moreover, given the increased transmissibility of Omicron variants, a higher percentage of individuals are likely to develop long COVID. Therefore, it remains crucial to elucidate the pathophysiological mechanisms and therapeutic options available to mitigate these clinical features [252]. A critical priority in Long COVID care is the early enrollment of patients into comprehensive rehabilitation programs to systematically identify and address treatable physical, emotional, cognitive, and social traits [257]. Proactive management of pre-existing comorbidities is to prevent clinical deterioration, minimize readmission rates, and stave off the development of secondary pathologies [257].

Currently, the emergence of novel SARS-CoV-2 variants and the risk of reinfection represent significant challenges for long COVID [258]. Even individuals who do not develop long COVID following an initial SARS-CoV-2 infection remain susceptible during subsequent infections [258,259]. Notably, reinfection can trigger de novo long COVID or exacerbate the severity of pre-existing long COVID [258]. This analysis indicates that the cumulative incidence of long COVID is significantly higher in cases of dual infection compared to single infection, and a third infection further increases this risk [19,258]. Therefore, given its high prevalence and diverse clinical presentations [260,261], LC is considered a major public health concern [262]. From a policy perspective, this condition places considerable strain on healthcare systems and national economies, hindering progress towards global health objectives such as the Sustainable Development Goals (SDGs) [19].

At present, investigating LC is critical; although the relative risk and mortality rate of PASC are lower in mild COVID-19 cases than in severe ones, the absolute number of mild cases is significantly larger [263]. Consequently, a significant population burden of PASC is driven primarily by mild infections. Data from the Global Burden of Disease (GBD) collaborators supports this, showing that approximately 90% of individuals diagnosed with PASC initially exhibited mild COVID-19. As a result, while preventing severe disease remains essential, mitigating the risk of post-acute and long-term health loss in mild cases is equally imperative [263]. Certainly, the elevated risk of death over 3 years among hospitalized patients with COVID-19 suggests that acute infection severity remains a pivotal factor in the manifestation of prolonged adverse outcomes [263].

To date, multiple drivers for long COVID syndrome have been proposed, including widespread blood inflammation and abnormal clotting, viral persistence in different organs, immune system dysregulation, mitochondrial dysfunction, neuronal inflammation and microbiome dysbiosis [264,265]. Indeed, inflammatory mediators arising from systemic inflammation may contribute to endothelial dysfunction, a hallmark of post COVID-19 syndrome [266] (Figure 4).

The clinical relevance of ED lies in its role as an independent risk factor for long COVID-19 [267], particularly concerning non-respiratory symptoms [268]. Emerging evidence suggests that endothelial damage and subsequent dysfunction may represent a common underlying mechanism for most post-acute complications, including arterial and venous thrombosis [28]. This pathological process may also be associated with an elevated risk of adverse cardiovascular events which can persist for at least 12 months following recovery from SARS-CoV-2 infection [269].

Numerous endothelial biomarkers and vascular function tests have been proposed to evaluate endothelial dysfunction in patients with long COVID [246,270,271]. Studies investigating the deleterious effects of SARS-CoV-2 on the systemic vasculature indicate impaired NO levels and diminished vascular function. This is evidenced by a reduction in the reactive hyperemia index (RHI) and flow-mediated dilation (FMD) measurements, which become apparent several weeks following a positive SARS-CoV-2 test result [246,272,273].

A prospective observational study demonstrated a strong correlation between endothelial dysfunction (indicated by FMD reduction three months post-acute phase) and the severity of acute COVID-19. This correlation remained consistent and independent of traditional risk factors for endothelial dysfunction [273]. Furthermore, the association between FMD and COVID-19 severity persists beyond the initial three-month period, a pattern distinct from that observed in C-reactive protein (CRP) measurements. The hypothesis proposed is that patients with moderate-to-severe COVID-19 exhibit a localized sub-inflammatory state restricted to the endothelium, which facilitates medium-term dysfunction without inducing systemic CRP production. Consequently, FMD may serve as a surrogate marker for persistent endothelial inflammation following SARS-CoV-2 infection, potentially identifying patients at elevated risk for post-acute COVID-19 syndrome [273].

Epidemiological data have indicated that individuals with underlying comorbidities such as diabetes, hypertension, heart failure, and coronary heart disease—as well as factors like smoking and advanced age—may be more susceptible to developing severe forms of COVID-19 [87,118,274,275,276]. These factors are collectively associated with pre-existing endothelial dysfunction. In these populations, COVID-19-related inflammation exacerbates endothelial damage. Indeed, it has been demonstrated that these patients exhibit a significantly diminished FMD, resulting from the synergistic effect of pre-existing dysfunction and COVID-19-induced inflammation [273].

One of the hypotheses underlying the development of ED in LC is that increased levels of ROS, reduced NO levels, and enhanced angiotensin II activity contribute to the progression of chronic hypoxia [277]. It has been demonstrated that this condition induces systemic physiological responses, including vascular remodeling, pulmonary fibrosis and hypertension. Subsequently, the combination of prolonged viral presence, chronic hypoxia, and persistent inflammation leads to sustained endothelial damage. This state is characterized by persistent coagulation activation, microvascular injury, and low-grade thrombus formation, which collectively drive systemic organ damage [102,278,279] (Figure 4) and, in some cases, arterial stiffness and declining lung function [280].

In patients with long COVID who exhibit a decline in RHI and FMD, there is an increased occurrence of acute coronary syndromes, acute pulmonary embolectomies, deep-vein thrombosis, and ischaemic stroke [272]. These conditions have been linked to the persistence of inflammation, the cytokine storm, microvascular damage, and stress-induced cardiomyopathy [80]. The prevalence of these findings is particularly notable among patients aged ≥75 years and those with dysmetabolic conditions or chronic kidney disease [281]. A similarly high prevalence is observed in individuals with a history of venous thromboembolism or coronary, carotid, or peripheral arterial disease [281].

Furthermore, evidence indicates that patients who have experienced an acute infection often present macrovascular and microvascular endothelial dysfunction and vascular stiffening several weeks to months later [282]. This delayed onset occurs even without a formal PASC/LC diagnosis and is notably pronounced in patients who lacked initial respiratory symptoms [282]. Irrespective of PASC, patients who have undergone COVID-19 exhibit impaired endothelium-dependent vasodilation and increased arterial stiffness, which may persist for at least 18 months following acute infection [282]. More importantly, Osiaevi and colleagues reported a reduction in vascular density and microvascular health scores in LC, indicating a potential long-term impact on the vascular system regardless of acute-stage severity. This suggests that microvascular impairment may play a significant role in both acute COVID-19 and post-acute sequelae [283].

It is evident that persistent endothelial dysfunction may trigger clinically relevant macro- and microvascular thrombotic events. Nevertheless, definitive clinical and experimental proof directly linking endothelial dysfunction to specific long-term symptoms is currently lacking. Further longitudinal studies are required to firmly establish these causal relationships and to ascertain the long-term impact of such endothelial dysfunction on health outcomes.

## 10. Folic Acid, Homocysteine and Vitamin B12 Metabolism in Long COVID

Consistent with acute COVID-19 observations, ED is associated with high Hcy levels in post-COVID-19 patients six months after SARS-CoV-2 infection, compared to healthy controls [266]. As previously noted, hyperhomocysteinemia (HHcy) is an important biomarker of impaired Folate-Mediated One-Carbon Metabolism (FOCM) [284]. Accordingly, in LC/PASC, HHcy may be a relevant factor in inducing oxidative stress via the increase in reactive oxygen and nitrogen species (RONS) in endothelial cells (ECs). This mechanism leads to ECs activation and dysfunction, which is detrimental to the normal functions of multiple tissues and organ systems [284].

The growing importance of HHcy in LC/PASC is underscored by the strong neurological symptom overlap between this chronic syndrome and other pathologies—such as myalgic encephalomyelitis (ME)/chronic fatigue syndrome (CFS)—which are all characterized by elevated levels of Hcy [285,286,287,288]. Moreover, it is now widely acknowledged that HHcy exerts vasculotoxic and neurotoxic effects. In the context of LC/PASC, this toxicity promotes neuronal inflammation, degeneration, and pro-oxidation, while activating proatherogenic and prothrombotic mechanisms [62,287,289].

Additionally, accumulating evidence suggests an independent association between HHcy and cognitive impairment [172,290]. The ‘brain-fog’ described by long-term COVID patients, along with cognitive deficits in post-COVID-19 patients [291], have been attributed to supranormal homocysteine levels [288]. Indeed, cognitive impairment and dementia are frequently correlated with elevated blood homocysteine [292]. Increased Hcy levels may induce both direct and indirect vascular damage and have been linked to vascular dementia and a higher risk of multiple brain infarcts [293].

Vitamin B12 is another critical biomarker that participates in the folate cycle and plays a relevant role in long COVID. As illustrated in Figure 2, vitamin B12 serves as a cofactor for the key one-carbon metabolism enzyme, methionine (Met) synthase (MS). This enzyme remethylates Hcy into Met, thereby linking the Met cycle with the folate cycle. Met, synthesized by MS, is subsequently used to produce S-adenosylmethionine (SAM)—a universal methyl donor for various acceptors, including those involved in epigenetic regulation [179] (Figure 2).

Notably, lower concentrations of folate and vitamin B12 were identified one year later in patients who had experienced a severe acute phase of COVID-19 [294]. These suboptimal values in LC/PASC patients are potentially related to the neurological symptoms commonly reported in patients with pernicious anemia—a condition secondary to vitamin B12 deficiency—where impaired methylation status predominates [179,284,288]. These symptoms include fatigue, memory complaints, sleep disturbance, numbness/tingling, confusion, dizziness, headaches, depression, gait disturbances, and hyposmia [288].

Furthermore, a moderate increase in all-cause mortality was observed in association with low serum B12 concentrations (defined as <140 pmol/L). These causes comprised, but were not limited to, chronic lower respiratory diseases, Alzheimer’s disease, influenza and pneumonia, cerebrovascular diseases, and diabetes mellitus. Concurrently, high serum concentrations of MMA (methyl malonic acid) and Hcy were also detected in these conditions [295].

The detrimental effects of inadequate vitamin B12 levels are also linked to endothelial dysfunction, as observed in other pathological contexts related to long COVID—including diabetes, atherosclerosis, myocardial infarction and stroke [296,297]. These associations support the hypothesis that vitamin B12 plays a critical role in LC pathogenesis.

In addition, the effects of vitamin B12 deficiency may be potentiated by the inflammatory microenvironment characteristic of long COVID. Patients experiencing chronic inflammation due to SARS-CoV-2 response are more susceptible to oxidative stress via increased RONS [284,287]. Therefore, under these oxidative conditions, vitamin B12 (cobalamin I) may undergo oxidation to cobalamin II, rendering it unable to mediate the MS reaction. Consequently, MS cannot convert Hcy into Met (leading to HHcy formation in the methionine cycle) or methyl-THF (tetrahydrofolate) into THF (in the folate cycle) [286,287,288,298] (Figure 2). These alterations in folate metabolism result in the hypomethylation of DNA, proteins, and lipids, ultimately leading to inflammatory damage and apoptosis within cerebrovascular structures and neurons [287].

As established by numerous studies, both folate and cobalamin (Cbl) are essential micronutrients within the central nervous system (CNS), and a positive correlation exists between their concentrations in plasma and cerebrospinal fluid (CSF) [299]. The clinical relevance of these biomarkers is particularly important in patients with LC, as it was previously reported that low plasma folate concentrations are associated with low CSF folate levels, a pattern consistent with neurodegenerative disorders [299].

Finally, MTHFR genetic variations also have a pivotal role in the pathogenesis of ED during LC. Emerging evidence suggests that the 677C>T variant is significantly correlated with increased disease severity [300] and this polymorphism is a critical marker for the cardiovascular and neurological complications [301] frequently observed in COVID-19 cohorts.

The clinical implications of MTHFR mutations are particularly evident in patients presenting with PASC-CVD [302]. This phenotype is characterized by abnormal myocardial strain and mechanical work, profound tissue perfusion deficits and impaired endothelial function [302]. In a documented case of PASC-CVD, the development of ED appears to be driven by a genetic predisposition (specifically the homozygous MTHFR C677T polymorphism) for a systemic prothrombotic state [302]. This pathology is further exacerbated by platelet hyperreactivity and post-COVID-19 syndrome, which is defined by a sustained, unresolved inflammatory response following the acute immunological phase [302]. The persistence of this inflammatory state is evidenced by chronically elevated anti-SARSCoV-2 IgG titers six months post-infection and vaccination [302].

Furthermore, cardiovascular magnetic resonance (CMR) imaging has revealed that myocardial inflammation, often manifesting as diffuse edema, interstitial expansion, and focal fibrotic remodeling, can persist even in patients with mild clinical presentations of COVID-19 and apparent recovery [302]. Notably, some studies suggest that the homozygous C677T mutation may confer a thrombotic risk profile comparable to that of chronic tobacco use [302].

The interplay between MTHFR polymorphisms and one-carbon metabolism is reported in a recent untargeted, longitudinal plasma metabolomics study in which one-carbon metabolism dysregulation is a hallmark of severe COVID-19 progression [303]. Integrating these metabolic profiles with patient genomics identified the C677T allele of the MTHFR gene as a critical determinant of clinical trajectory [303]. Specifically, patients homozygous for the C677T variant exhibit profound metabolic aberrations that correlate with a significantly higher incidence of severe disease. These data suggest that MTHFR genotypic status may serve as a cornerstone for individualized therapeutic interventions [303].

Beyond alterations in one-carbon metabolism, disruptions in DNA methylation play a pivotal role in the pathophysiology of long COVID. Systematic identification of distinctive differentially methylated regions (DMRs) in LC patients has permitted the differentiation from healthy controls, while simultaneously providing a molecular framework for disease stratification based on clinical severity [304]. These epigenetic signatures underscore the diagnostic potential of DMRs as robust clinical biomarkers [304]. Furthermore, these data suggest that genetic variation within *MTHFR* influences long COVID symptoms by modulating key methionine cycle metabolites, such as homocysteine [303]. Reduced *MTHFR* activity correlates with elevated homocysteine levels, a condition that promotes endothelial dysfunction and systemic inflammation [305]—mechanisms underlying the progression of long COVID.

Collectively, these findings illustrate a complex interplay between epigenetic reprogramming and metabolic dysregulation in long COVID, identifying a critical axis that may dictate disease trajectory. Further investigation into the longitudinal stability of these signatures and their causative role in disease progression is warranted, as this mechanistic insight could facilitate the development of targeted therapeutic strategies to mitigate long-term complications.

The clinical application of *MTHFR* genotyping and plasma metabolomics at the point of admission offers a robust framework for early risk assessment, as these biomarkers are informative during the initial hospital visit [303]. By synthesizing genetic and metabolite data into a multimodal predictive model, clinicians can achieve early patient stratification and tailor medical recommendations to optimize acute care and mitigate long-term complications.

## 11. Folic Acid and Vitamin B12 Administration in Long COVID

Considering the comparatively reduced levels of vitamin B12 and folate identified in LC/PASC, several authors have proposed supplementation as a complementary treatment. For example, Regland et al. demonstrated that individuals suffering from illnesses with symptoms analogous to LC/PASC, such as chronic fatigue syndrome/myalgic encephalomyelitis (CFS/ME) and fibromyalgia, responded favorably to B12 injections and oral folic acid, resulting in symptom mitigation [285].

Moreover, the use of anti-inflammatory therapy (such as methylprednisolone) in conjunction with micronutrients supplements that support one-carbon metabolism—specifically vitamin B12 and B9–folic acid—has been proposed to treat neurological symptoms [287]. Principally, this therapy could help to suppress the chronic peripheral pro-inflammatory cytokines/chemokines (PCC) and the central nervous system cytokines/chemokines (CNSCC) neuroglia, neuroinflammation, and impaired cognition, all of which contribute to LC/PASC syndrome [287]. This is particularly significant as evidence suggests that, irrespective of acute COVID-19 severity, both younger and middle-aged individuals demonstrate a disproportionate prevalence of Neuro-PASC.

While abnormal neurological findings and comorbidities are more prevalent among the elderly, younger and middle-aged patients frequently present with a higher prevalence of Neuro-PASC symptoms and cognitive dysfunction, significantly impacting their quality of life [306]. Notably, in young patients diagnosed with mild cognitive impairment (MCI), FA has demonstrated both anti-inflammatory and memory-enhancing properties, representing a viable therapeutic option. Following a twelve-month regimen of 400 μg of FA daily, subjects showed notable enhancements in cognitive performance and reduced systemic inflammation, evidenced by a decline in peripheral inflammatory cytokine levels [307]. In addition, recent reports indicate that FA plays a pivotal protective role in chronic inflammatory pain, making it a promising candidate for neuroinflammatory disease treatment due to its significant neuroprotective properties [308].

Latest findings identified the role of FA in a high-resolution profiling study of immune cell populations within the frontal lobe, which has elucidated the complex landscape of immune dysregulation and synaptic impairment driving cognitive decline in COVID-19 patients [309]. By characterizing these neuroinflammatory signatures, the authors confirmed pivotal genetic regulators that may serve as both diagnostic biomarkers and therapeutic targets for COVID-19-associated cognitive impairment [309]. In this drug repurposing analysis, folic acid has been identified as a candidate compound for treating COVID-19-associated cognitive decline, further highlighting its therapeutic potential in clinical management [309].

In conjunction with FA, the role of vitamin B12 was further investigated in a multicenter, randomized, placebo-controlled trial. The results substantiate the efficacy and safety of prednisolone combined with vitamins B1, B6 and B12 in alleviating LC symptoms [310]. Furthermore, it has been reported that high-dose vitamin B supplementation similarly reduces Hcy levels, slows cognitive decline, and mitigates brain atrophy [311], suggesting a neuroprotective role in Alzheimer’s disease [312] and a potential preventive strategy for LC/PASC. Specifically, the reduction in homocysteine levels via elevated doses of B vitamins appears to drive these neuroprotective effects. However, the clinical efficacy of this intervention is contingent upon baseline homocysteine concentrations present in the subject. Additionally, metabolic evidence of vitamin B deficiency should be confirmed prior to initiating the protocol to ensure optimal outcomes [313].

It is important to note that pharmacological treatment with B12 generally requires high doses—typically 1000–2000 μg/d—over a duration of 1–3 months [314,315]. Moreover, the therapeutic potential of vitamin B12 extends beyond the management of hyperhomocysteinemia-related vascular disease [299]; it offers symptomatic relief that may improve clinical prognosis during and after the acute phase of COVID-19 syndrome.

Taken together, therapeutic intervention with vitamin B12 demonstrates efficacy in mitigating oxidative damage and inflammatory responses within both systemic and central neurological systems. This neuroprotective profile is particularly enhanced when administered alongside FA supplementation. Recent studies indicate that combined FA and B12 treatment reduces pro-inflammatory cytokines and modulates inflammatory pathways in neuropathic pain—a condition that may also manifest in post COVID-19 cases [316]. Consequently, vitamin B12 has been proposed as a complementary therapy for mild to severe COVID-19 symptoms, owing to its analgesic properties and its role in managing neuromuscular disorders prevalent in LC patients [317]. Thus, vitamin B12 supplementation, integrated with clinical nutrition, represents a potential adjuvant therapy to normalize HMRD (human metabolic reprogramming and dysregulation) in patients with COVID-19 and PASC [6].

In this context, vitamin B12 is a key modulator of immune responses, predominantly exhibiting anti-inflammatory properties and therapeutic potential in inflammatory and neuroimmune-related conditions [316]. For instance, administration of vitamin B12 effectively mitigates the pro-inflammatory transcriptional profile in leukocytes from patients with moderate to severe COVID-19, with no reported adverse effects [318]. Specifically, vitamin B12 downregulates inflammatory genes and metabolic signaling pathways associated with COVID-19 via epigenetic mechanisms mediated by DNA methylation [318]. In hospitalized patients with severe COVID-19, vitamin B12 reduces the hyperinflammation [318], suggesting its utility as an adjuvant therapy when combined with established treatments.

Regarding inflammation management, studies have demonstrated a positive correlation between higher vitamin B12 levels and lower levels of Interleukin-6 (IL-6) [319], a key mediator in the progression of chronic and acute inflammatory processes, including long COVID [320]. Furthermore, vitamin B12 offers antioxidant properties, an excellent safety profile, and high cost-effectiveness.

More recently, it was reported that vitamin B12 can epigenetically modulate the expression of the chemokine CCL11—which affects microglia, astrocytes, and neural progenitor cells in the CNS—in patients with LC [321]. Increased levels of CCL11 were correlated with a LC cognitive symptom, the visuoconstructive deficit (VCD), observed in young patients following mild acute COVID-19 [321]. This cognitive symptom is characterized by an impaired ability to visually perceive and construct complex visual–spatial designs or tasks and is a well-recognized feature of aging-related neurodegenerative diseases, including Alzheimer’s disease.

In the current study, patients exhibited signs of systemic inflammation alongside elevated levels of the chemokine CCL11 in peripheral leukocytes. Vitamin B12 shows potential as an epidrug, capable of enhancing CCL11 methylation and subsequent downregulation in peripheral leukocytes from individuals diagnosed with VCD [321]. Indeed, supplemental vitamin B12 holds promise for addressing other neurodegenerative conditions linked to aberrant CCL11 expression, such as LC, thereby preventing or mitigating LC-associated VCD. This study paves the way for future research into the role of vitamin B12 in regulating inflammatory markers elevated in cognitive syndromes.

Finally, it is noteworthy that the beneficial effects of FA and vitamin B12 treatment in the context of LC extend beyond neurological symptoms. A recent report indicated that FA and B-complex vitamins complement pharmacological treatment for angina control in a young woman with post-COVID-19 syndrome [302]. This precision-guided therapeutic regimen incorporated tailored antianginal agents coupled with an individualized antithrombotic strategy. Specifically, the treatment consisted of long-term folic acid and vitamin B complex supplementation—targeted to the patient’s MTHFR mutation—combined with low-dose aspirin for the management of sticky platelet syndrome [302].

At one-year follow-up, multimodality imaging revealed significant myocardial recovery, characterized by the near-normalization of tissue parameters [302]. These objective findings correlated with a robust clinical response, as the patient reported a substantial reduction in both the frequency and intensity of anginal symptoms. Notably, the resolution of myocardial inflammation and edema served as a primary indicator of structural restoration and treatment efficacy, providing a clear pathophysiological basis for the observed clinical improvement.

It is important to note that FA dosage and treatment duration are vital for observing measurable changes in LC symptoms. As reported in a double-blind, placebo-controlled, randomized clinical trial involving patients with mild COVID-19, the administration of a micronutrient supplement containing 400 µg of FA for just 14 days did not prevent PASC symptoms during the six-month post-treatment follow-up [322]. These findings underscore the necessity of optimizing treatment protocols for both FA and vitamin B12 in the management of LC.

In summary, further randomized controlled trials and analysis of vitamin B12 and folate metabolism markers—such as total B12, holotranscobalamin, total Hcy and MMA, total folic acid, polymorphism and/or methylation of genes—are required to standardize administration protocols [317]. A deeper understanding of dysregulated one-carbon metabolism in COVID-19 suggests that supplementation with vitamin B12 and folic acid may benefit patients experiencing LC/PASC-related impairments. Ultimately, insights gained from defining the biology and treatment of long COVID may offer therapeutic implications for other populations living with IACCs (infection-associated chronic conditions) [323].

## 12. Conclusions

While vaccination and pharmacological strategies have reduced COVID-19-related mortality, significant challenges remain in preventing primary infection and the development of acute and long-term symptoms. Consequently, a significant number of patients worldwide still require hospitalization or suffer from chronic complications of long COVID. Since the beginning of the pandemic, the efficacy of nutraceuticals has been explored as potential therapies to prevent disease onset or improve clinical outcomes in both the acute phase and the management of chronic sequelae. In this review, the role of folate metabolism was discussed within the context of endothelial dysfunction characteristic of COVID-19 patients.

Since folic acid supplementation is a safe and accessible treatment for various pathologies, increasing FA intake alongside vitamin B12 could be considered as a complementary intervention to improve the prognosis of patients with mild or severe presentation of COVID-19 and LC. Furthermore, considering its benefits for endothelial health, regular folic acid supplementation may serve as a preventive therapy, particularly for vulnerable patients at high risk of developing acute or long COVID-19. However, while oral supplementation of FA has been proposed as a potential strategy to mitigate ED, it is critical to note that this hypothesis is currently supported primarily by cross-sectional data, and clinical trials evaluating FA specifically in long COVID are limited.

Additionally, while a high dose (defined here as >5 mg/day) is often discussed in the literature, potential adverse effects such as pregnancy complications, offspring disease risk, neurodevelopmental deficits, altered immune/allergic responses and accelerated carcinogenesis must be carefully weighed. Therefore, robust clinical trials are urgently needed to establish both safety and efficacy before clinical implementation can be recommended. Importantly, monitoring Hcy levels in COVID-19 patients with hyperhomocysteinemia is necessary to evaluate disease progression and the efficacy of folic acid treatment.

It is clear that further large-scale intervention studies are needed to assess the efficacy of FA in COVID-19 prevention and treatment. Given the evidence presented here, folic acid research deserves more attention than it has received to date. Specifically, double-blind, placebo-controlled randomized clinical trials are required to evaluate the role of FA in acute COVID-19 and its potential impact on long COVID. As patient diversity is crucial, recruitment must include participants from various racial and ethnic backgrounds to ensure the broad clinical applicability of novel procedures.

Finally, the mechanisms underlying COVID-19-induced endothelial dysfunction and long-term effects on vascular function require thorough investigation. Furthermore, it remains to be determined whether COVID-19 contributes to atherosclerosis and inflammatory vascular diseases over time. A better understanding of this relationship may lead to therapeutic interventions that reduce COVID-19 severity in the acute phase and mitigate the long-term syndrome, considering that the effects of LC may accumulate with multiple reinfections.

Thus, further research is necessary to determine whether targeting these metabolic pathways holds therapeutic potential to prevent not only COVID-19 but also other viral diseases with long-term symptoms.

## Data Availability

No new data were created or analyzed in this study. Data sharing is not applicable to this article.
